# Open-Phylo: a customizable crowd-computing platform for multiple sequence alignment

**DOI:** 10.1186/gb-2013-14-10-r116

**Published:** 2013-10-22

**Authors:** Daniel Kwak, Alfred Kam, David Becerra, Qikuan Zhou, Adam Hops, Eleyine Zarour, Arthur Kam, Luis Sarmenta, Mathieu Blanchette, Jérôme Waldispühl

**Affiliations:** 1 School of Computer Science and McGill Centre for Bioinformatics, McGill University, McConnell Engineering Bldg, Rm 318, 3480 University Street, Montreal QC H3A 0E9, Canada; 2 Microsoft, 1020 Enterprise Way, Sunnyvale, CA 94089, USA

## Abstract

Citizen science games such as Galaxy Zoo, Foldit, and Phylo aim to harness the intelligence and processing power generated by crowds of online gamers to solve scientific problems. However, the selection of the data to be analyzed through these games is under the exclusive control of the game designers, and so are the results produced by gamers. Here, we introduce Open-Phylo, a freely accessible crowd-computing platform that enables any scientist to enter our system and use crowds of gamers to assist computer programs in solving one of the most fundamental problems in genomics: the multiple sequence alignment problem.

## Background

Multiple sequence alignment (MSA) algorithms are among the most powerful tools available today to study the evolution and function of DNA, RNA and protein sequences [1]. Key to these analyses is the ability to align multiple sequences accurately, a computationally hard problem [2]. Over the last 40 years, computational methods have considerably improved, to a point where fairly reliable alignments of multiple complete genomes are now feasible [3]. Nonetheless, such alignments often contain local inaccuracies and benefit from manual curation and fine-tuning. Further, popular alignment databases such as Rfam [4] are now semi-automatically collecting improved alignments submitted by their users.

Recently, we introduced Phylo [5,6], a casual online puzzle, which translates small-scale multiple sequence alignment problems into puzzles, whose solutions, produced by online gamers, are used to improve the accuracy of MSAs obtained with state-of-the-art alignment algorithms. Importantly, Phylo is a purely ludic game that can be played by untrained web users with almost no prior knowledge of the biological context. This unique feature enables it to reach a broad audience ranging from teenagers to seniors, and casual gamers with a short gaming time and attention span.

As with many other crowdsourcing platforms, such as Galaxy Zoo [7], Foldit [8], EteRNA [9], Dizeez [10] and Eyewire [11], Phylo aims to harness the intelligence and processing power generated every day by crowds of online gamers. However, in all these cases, human computing power is placed at the service of the small group of researchers who formulated the problems to be solved by the crowd. The work proposed here aims to address this issue and to propose a new model for human-computing platforms, one that is powered by the public and is open for the public.

In this paper we introduce Open-Phylo [12], an open and freely accessible web interface that enables scientists to enter their own sequences into our system and manage the efforts of the crowd toward aligning them. In addition, we developed an advanced version of the game [13], where advanced players can play with larger MSAs, up to 300 nucleotides long. This allows us to benefit from the skills of the most experienced users more efficiently in solving the hardest MSAs.

We used Open-Phylo to align sequences from the promoter regions of three key cancer genes (the P53 tumor suppressor protein, breast cancer type 1 susceptibility protein (BRCA1), and retinoblastoma protein (RB1)). We show that crowds of gamers, managed through Open-Phylo, consistently improved the alignments computed using any state-of-the-art methods such as Multiz [14], MUSCLE [15], PRANK [16] and T-Coffee [17]. Here, we show (i) that most alignments calculated by computer programs can be improved by gamers and (ii) that a large group of casual players provide a processing power that can outperform the work produced by smaller numbers of advanced players.

## Results and discussion

### An open crowd-computing system

**Figure 1:**
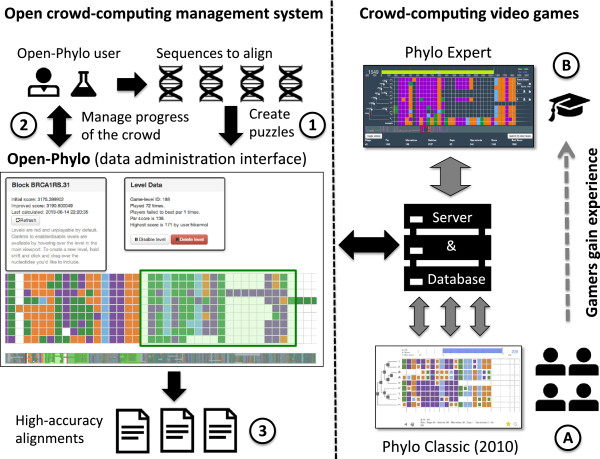
**Open-Phylo crowd-computing system. (1)** Scientists upload their sequences to the database, validate the alignment puzzles built by the system (See green box in the data administration interface), or select new ones. **(2)** The same users monitor the progress of the crowd in improving their alignments, close puzzles, open new puzzles and finally **(3)** download the best solutions. The crowd-computing engine is powered by **(a)** many casual gamers playing classic puzzles and **(b)** a smaller number of experienced players, who have access to larger and more difficult puzzles.

Open-Phylo is the first crowd-computing system that is open for the benefit of the whole scientific community. It uses the processing power generated by video gamers (Figure 1). At the first glance, Open-Phylo looks like a traditional web server. Users are asked to register to access our interface and upload their sequences. Input sequences are first aligned using one of the publicly available algorithms, forming the initial configuration for the crowd-based work that follows. Once the sequences are entered into our database, the submitter accesses the *crowd manager*: a private interface that implements the tools to manage and monitor the data. In particular, the workspace lets users identify, either automatically or manually, portions of the alignment on which the crowd-based improvements should be focused, that is, those for which puzzles, either short 'casual’ 20-column subalignments or longer 'expert’ 300-column subalignments, should be generated. The submitter can track in real time the number of times each puzzle has been played and the magnitude of the improvement to the alignment score achieved by the crowd. At any time, the user can remove from the pool puzzles that he/she feels have been played sufficiently often, or to add new ones. This functionality allows the submitter to manage the work of the crowd. Crowd-improved MSAs can be downloaded at any time.

Although Open-Phylo is based on a player interface similar to Phylo’s, it features several key innovations that significantly broaden its player base, including support for mobile devices such as most popular tablets and browsers, and social-login and social-share capabilities allowing easier logging in and improving the sustainability of the crowd. More importantly, the new crowd-computing system also features a new expert gaming interface (Figure 1), which allows the most experienced users (who have completed at least 20 puzzles) to play much larger puzzles (MSAs up to 300 nucleotides long). This latter feature enables us simultaneously to motivate the best players to keep playing the game and to use more efficiently the skills developed by dedicated players.

The Open-Phylo submission interface has several key functions. First, users can select the objective function for identifying the best alignments. In addition to classical scoring functions such as Ancestor [18], MUSCLE [15] or T-Coffee [17], users can also directly select the best alignments calculated by the players with the scoring scheme used in the games (that is, the highest scoring puzzles in the video game). Next, submitters can intuitively create casual puzzles using the GUI by selecting an area of the MSA. Finally, submitters can create a personal profile and provide a brief overview of their research. These profiles are accessible to Phylo’s players and are intended to promote the research conducted by the participating scientists and to initiate communication and knowledge transfer between the geneticists and the player community.

Task routing is important for ensuring the efficiency of human-computing systems [19]. In the classic version of Phylo, we implemented, a priority queue based on the number of times a puzzle has been played. Puzzles with few solutions have a high priority. A different mechanism has been implemented in the expert version, which uses a pull approach and users can decide which puzzles they wish to play. The expert version has a menu that shows all available puzzles together with statistics for each of them (including the number of times a particular expert puzzle has been played, its base score and current high score). Users can search and sort this menu to find the most interesting and promising expert puzzles to play. This system aims to benefit from the experience of advanced players in identifying puzzles that need the most work from the player community. Moreover, the expert version also allows users to play puzzles that have already been improved by other players. This feature allows collaborative work and potentially increases performance.

### Case study and performance

To illustrate and evaluate the alignment capabilities of Open-Phylo, we used it to align sets of orthologous promoter sequences (regions of 1,000 bp located upstream of the transcription start site) of three key cancer genes from 12 different species of mammals. Each set of orthologous promoter sequences was initially aligned using one of four state-of-the-art algorithms: Multiz [14], MUSCLE [15], PRANK [16] or T-Coffee [17]. The resulting MSAs ranged in size from 1,222 to 3,346 columns. For each initial MSA, we used Open-Phylo’s crowd-computing management system to direct the crowd efforts to a set of 79 (overlapping) expert-level puzzles of 300 alignment columns each. From the MSAs calculated by each of the four alignment programs, 1,014 casual-level puzzles (20 nucleotides long) were extracted and these were used as initial configurations for the levels of the casual game (also referred to as the classic game). Whereas solutions to expert-level puzzles can be directly evaluated using a given objective function (see below), solutions to casual-level puzzles need to be reinserted into the larger alignment context before they can be scored.

**Figure 2:**
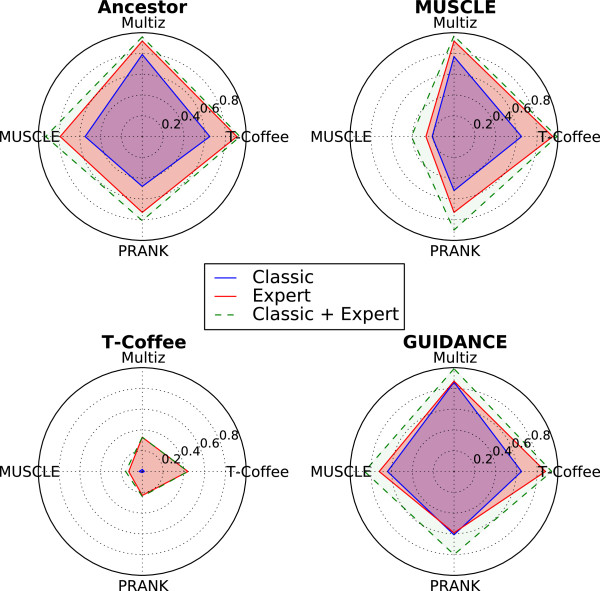
**Performance of Open-Phylo using the casual or expert version of the Phylo video game.** Ratio of puzzles improved by Open-Phylo for the scoring functions Ancestor (top left), MUSCLE (top right), GUIDANCE (bottom right) and T-Coffee (bottom left). The alignment program used to calculate the initial MSAs is indicated on the axis of the radar charts: Multiz (north), MUSCLE (west), PRANK (south) and T-Coffee (east). The area surrounded by a blue line corresponds to the performance achieved with the casual puzzles only, while the area surrounded by a red line indicates the performance of the expert version only. The area surrounded by a dashed green line shows the ratio of alignments improved by either the classic or expert version.

Between 3 December 2012 and 3 April 2013, 12,961 unique visitors proposed solutions for 1,352 puzzles, including 338 expert-level and 1,014 casual-level puzzles. We assessed the extent to which the quality of an MSA could be improved through Open-Phylo. There is no single well-accepted scoring scheme for MSAs and each of the four aligners considered uses a different objective function. We thus evaluated each of the MSAs obtained using each of the following four scoring functions: Ancestor (a likelihood score reflecting both substitutions and indels on a given tree, which is approximated by the scoring function that Open-Phylo players try to optimize) [18], MUSCLE, GUIDANCE (a program that calculates the confidence score used by PRANK) [20] and T-Coffee. We evaluated the percentage of the 338 alignment blocks whose score was improved through Open-Phylo (either in casual or expert mode), starting from the alignments produced by Multiz, MUSCLE, PRANK or T-Coffee, and using each of the four scoring functions (Figure 2). More precisely, we evaluated all solutions submitted by casual gamers and advanced players and kept only the best for each objective function. Our experiments revealed that, depending on the objective function used, Open-Phylo improved 32% to 97% of Multiz alignments, 16% to 93% of MUSCLE alignments, 24% to 90% of PRANK alignments and 43% to 99% of T-Coffee alignments. In practice, our data suggest that the top 40% of casual solutions, ranked using the game scoring function, are sufficient to reproduce our results (see section 'Improvement of MSA with casual levels’).

Open-Phylo appears to have the potential to improve a significant fraction of alignments calculated by any method for any scoring function. We obtained the largest improvements with the Ancestor and GUIDANCE scoring functions. Interestingly, these functions are precisely those that use the same user-defined phylogenic tree to score an alignment as the game. In both cases, and also for the MUSCLE objective function, we observed that for up to 62% of the cases, the solutions calculated from casual puzzles outperform those submitted by experts. This suggests that the work of many casual gamers can in some cases compensate for the lack of experts. Casual gamers are an important processing resource, who should not be neglected. However, this might not be the case for alignments calculated with T-Coffee, as the 44% improvement (using the T-Coffee objective function) was obtained almost exclusively from expert submissions. This discrepancy could be explained by the differences between the scoring scheme used in T-Coffee and the one used by our game. Nonetheless, since the latter achieved satisfactory performance with all other programs as well as with the T-Coffee objective function using the expert submission, we consider that the scoring scheme used in the game provides reasonable performance.

**Figure 3:**
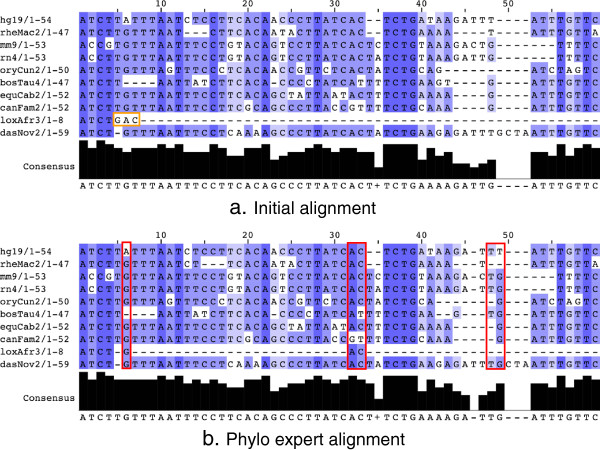
**A multiple sequence alignment improved with the expert version of Phylo. (a)** A section of the input alignment of the *P53* gene calculated with MUSCLE. **(b)** The improved alignment obtained with the expert version of Phylo. Three nucleotides from the elephant sequence (loxAfr3) have been moved to increase the conservation of alignment columns 6, 32 and 33. The player also improved the alignment of columns 48 and 49 and revealed similarities not found in the original alignment. Image produced with Jalview [21].

Overall, the magnitude of the improvement of the score is modest (Additional file 1: Table S1). For the classic version, the score improved by +1.9% (using the ANCESTOR objective function on MSAs calculated with Multiz), +28.4% (GUIDANCE/PRANK), +1.7% (MUSCLE) and 1.5% (T-Coffee). The expert version produced slightly larger improvements: +3.3% (ANCESTOR/Multiz), +10.9% (GUIDANCE/PRANK), +3.7% (MUSCLE) and 1.9% (T-Coffee). These values may appear low but are significant if we consider that the alignments calculated by the computers are already very well optimized. It also suggests that the players’ solution remains in the vicinity of the initial MSA. Nonetheless, even if the magnitude of the improvement is not very large, some alignments may present significant qualitative improvements. An illustration of such a case is shown in Figure 3. Here, an alignment of a portion of the promoter of the *P53* gene originally produced by MUSCLE was improved by an expert player, resulting in an increase in the alignment score from 3,533 to 3,906, and clearly improving the conservation of several alignment columns.

### Comparison of classic vs expert games

**Figure 4:**
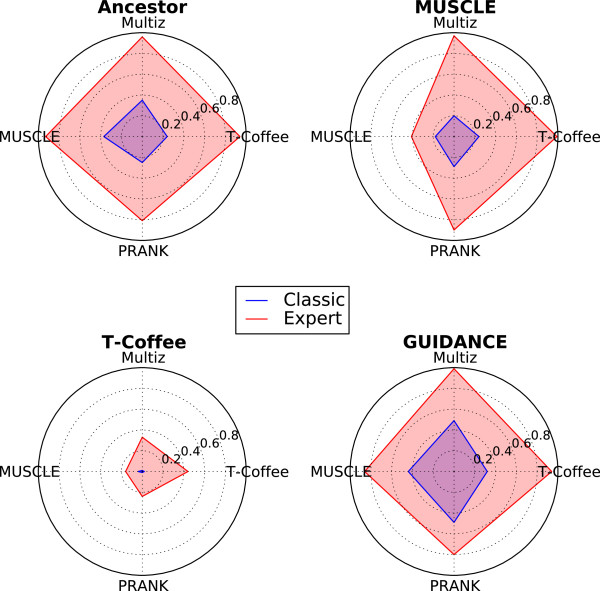
**Comparison of the improvements provided by the casual and expert versions.** The ratio of optimal solutions obtained with the casual version is shown in the area surrounded by a blue line, and the ratio obtained with the expert version in red. Each radar chart corresponds to one of the objective functions: Ancestor (top left), MUSCLE (top right), GUIDANCE (bottom right) and T-Coffee (bottom left). The alignment program used to calculate the initial MSAs is indicated on the axis of the radar charts: Multiz (north), MUSCLE (west), PRANK (south) and T-Coffee (east).

To better understand the relative performances of the classic and expert versions, we compared the results of the casual (classic) game to the results from the advanced player (expert) version. In particular, we investigated whether the classic or the expert version of the game provided the best improvement. We show these data in Figure 4. For each objective function (Ancestor [18], MUSCLE [15], GUIDANCE [20] and T-Coffee [17]) and each data set (initial MSAs computed with Multiz [14], MUSCLE [15], PRANK [16] or T-Coffee [17]), we determined which method (that is classic or expert) provided the highest improvement. The areas plotted in the radar charts correspond to the percentage of alignments improved by either the classic or expert version of Phylo, which are also plotted in green in Figure 2. When we normalized the data, we observed that 34% to 62% of the best solutions were produced by the classic version using GUIDANCE as a scoring function. At the other end of this spectrum, 76% to 96% of the best solutions were generated by the expert version with T-Coffee. Ancestor and MUSCLE provided intermediate results with, respectively, 26% to 40% and 20% to 43% of optimal solutions calculated with the classic version of the game. These data suggest that (i) casual gamers might provide a processing power that should not be neglected and (ii) the performance of the classic version depends on the objective function used by the Open-Phylo MSA submitter.

### Improvement of MSA with casual levels

All solutions generated by gamers for casual puzzles with a score (using the scoring scheme of the game) higher than or equal to the score of the initial level are stored in our system. We have to find those that provide the best improvement (if any) from the initial levels. Since the scoring function used in the game is not identical to the objective function we wish to use to select the best alignment (for example, Ancestor, MUSCLE, GUIDANCE or T-Coffee), we inserted all of the proposed solutions into their original location in the full MSA and evaluated the global improvement using the desired objective function.

**Figure 5:**
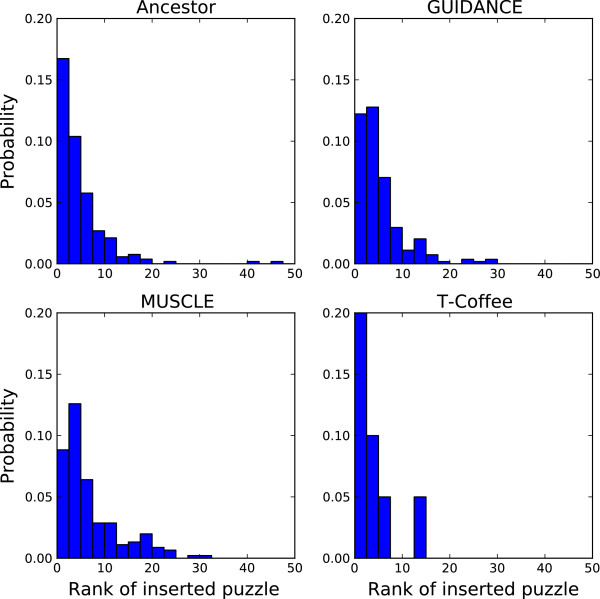
**Performance of the game scoring function in identifying the best alignments.** The graphs show the distributions of the rank (calculated using the scoring function used in the game) of the best solutions found in the casual or classic game (that is where casual submissions were inserted into the initial MSA and found to have the best score). Each histogram corresponds to a different objective function: Ancestor (top left), GUIDANCE (top right), MUSCLE (bottom left) and T-Coffee (bottom right).

The performance of the human-computing system was thus determined by the agreement between the scoring scheme used in the game and the values returned by the objective function used to identify the best alignments. To evaluate this correlation, we plotted (Figure 5) the distributions of the rank (based on the scoring scheme of the game) of the inserted solutions (for the submissions providing the best improvement). Our data reveal that 98% of the best alignments belong to the top 20 best ranked solutions (using the game scoring scheme). On average, we collected approximately 40 to 50 solutions for each casual puzzle. This suggests that instead of trying to insert all submissions, we need only consider the top 40% of solutions for improving the initial MSAs, while keeping the same performance and saving time and processing power.

### Usage statistics

Between 3 December 2012 and 3 April 2013, 12,961 unique visitors (for a total of 22,713 visits) submitted 49,875 solutions for classic and expert puzzles, comprising 2,005 solutions for expert-level and 47,870 solutions for casual-level puzzles. During this period, in addition to the expert and casual puzzles (P53, BRCA1 and RB1 alignments) used for the benchmark, our database also included 56 other expert puzzles and 1,066 casual puzzles unrelated to our cancer gene test set, built from UCSC genome browser reference alignments.

We collected at least three solutions for each large MSA played in the expert version of Phylo. Of the puzzles, 7% to 27% (respectively, for the PRANK and MUSCLE data sets) were played more than five times. Thus, if we expect enhanced performance from the expert version, we must also expect a lower coverage (or lower submission rate) than with the casual version.

**Figure 6:**
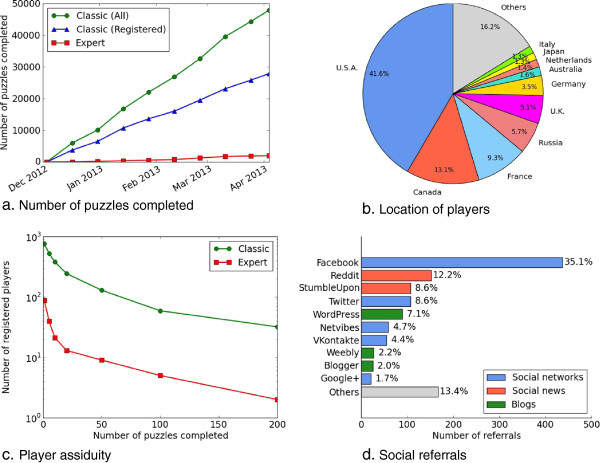
**Usage statistics for Phylo from 3 December 2012 until 3 April 2013. (a)** Number of classic (in green) and expert puzzles (in red) completed over time. The number of classic puzzles submitted by registered players is shown in blue. **(b)** Number of visits to the Phylo website per country. **(c)** Number of registered players vs minimum number of puzzles completed. Statistics for the classic version are shown in green for the classic version and in red for the expert version. **(d)** Social posts that led to a visit to Phylo. Social networks are shown in blue, social news services in red and blogs in green.

Figure 6 provides more details of usage statistics and user profiles. First, we observe in Figure 6(a) that the number of puzzles completed over time grew linearly with an approximate growth rate of 409 puzzles per day for the classic version. These results differ from those obtained for the original version of Phylo [5], which had a higher growth rate in the first weeks after the release of the game, due to the media coverage that followed the first release. In contrast, the games presented in this paper did not benefit from this media coverage. Interestingly, more than 2 years after its launch, our model seems relatively robust with a stable community of gamers. The same figure supports our original observation that guest users (those who did not register) generate approximately half of the solutions for classic puzzles. This result suggests that registration is not required. As anticipated, the number of solutions collected for expert puzzles is much lower than for classic puzzles. However, because the expert version is a recent addition to the Phylo system, this number could significantly increase in the coming months. Considering this lower number of submissions, the performance achieved by experts is very satisfying and supports the architecture of our system: the classic version can be used to train and identify users with the best skills after which they can participate more intensively.

Figure 6(b) shows the countries of origin of the players. Currently, it appears that the USA achieves the highest participation and that 75% of visits originated from five countries (the USA, Canada, France, Russia and the UK). The translation of the games into eight new languages (simplified and traditional Chinese, Hebrew, German, Portuguese, Russian, Romanian and Spanish in addition to English and French) is intended to improve the distribution of contributing countries in the future.

Figure 6(c) shows the contribution of individual registered players. As in our first analysis of usage statistics [5], we observe that most players play between one and ten puzzles. Of the 755 registered players, 525 completed more than 5 classic puzzles. Moreover, 242 players completed 20 puzzles and were allowed to enter the expert version, which 88 did. Registered players completed a total of 27,892 classic puzzles (37 puzzles per player on average), whereas non-registered players (identified only by their IP addresses) completed an average of only 1.6 puzzles. Finally, players who reached the expert level were quite assiduous, playing an average of 23 expert puzzles each. Notably, two players completed more than 200 expert puzzles each.

Figure 6(d) shows the impact of social recommendation on participation. Facebook provided the highest number of recommendations that led to a visit to the Phylo website. Overall, social networks (Facebook, Twitter, Netvibes, VKontakte and Google+) were the main source of traffic arising from social media. However, interestingly, if we ignore Facebook, social news services (Reddit and StumbleUpon) appear to provide the largest number of visitors. This observation suggests that communication strategies using these media are likely to have a substantial effect on the popularity of citizen science games.

## Conclusions

Our results suggest that humans can provide insights that cannot be entirely replicated by heuristics-based algorithms. This performance is most likely due to the capacity of humans to use their (visual) intuition to explore promising but abstruse configurations neglected by the heuristics implemented in alignment software.

Interestingly, we also observed that the scores of the best solutions from the four different initial alignments rarely converged to the same (or even similar) scoring alignments, suggesting that the players’ solution remained in the vicinity of the initial MSA. Indeed, even if two different scoring functions agree on the global features of the “best” MSAs, it is very unlikely that they will have the same global optima for all MSAs. Therefore, the performance of the system seems to be significantly influenced by the choice of the initial configuration, thus by the alignment program chosen by the submitter. Nonetheless, our results also suggest that Phylo is able to improve alignments for the most popular objective functions.

Open-Phylo is the first open-science platform that enables any scientist in the world to benefit from crowdsourcing and human-computing technologies to help in solving one of the most fundamental and widely used problems in bioinformatics. We believe that Open-Phylo is a pioneer for the next generation of crowdsourcing frameworks in biology: human-computing tools will be run by the people for the people.

## Materials and methods

### Selection of casual puzzles

Once uploaded in our system, levels (that is casual puzzles of 20 nucleotides used in the classic/casual version of the game) are extracted from the MSAs submitted by Open-Phylo users. The protocol used to determine the levels (automatically) is:

1. Pan a reading frame of ten to twenty nucleotides across the sequences.

2. Calculate the number of nucleotides (without gaps) for each species. Then, compute the average and standard deviation.

3. Calculate the number of pairwise matches between nucleotides in columns (ignoring the tree structure). From this number, derive the ratio of matches vs all possible pairwise comparisons within columns.

4. The level is accepted if the standard deviation in Step 2 is greater than 1, and the level match ratio/fraction in Step 3 is between 0.32 and 0.38.

5. If accepted, the reading frame jumps past the current nucleotides (to prevent an overlap). Otherwise, it shifts by one position to the right.

In addition, users can also create their own levels through the Open-Phylo web interface. To do so, a user selects a region (using the shift key) of the MSA with a size of between ten and twenty columns. All non-empty rows (that is with at least one nucleotide) are included in the new level.

### Advanced player (expert) version

We developed a version of Phylo for advanced players [13]. This interface is accessible to any registered user of the classic/casual version who has completed at least 20 puzzles (that is, they have reached the final stage of the game). It features several major upgrades:

• The game can display sequences with up to 300 nucleotides on a grid with 400 columns. As in the classic/casual version, the game can display 12 sequences instead of the 8 in the 2010 version of the casual game Phylo.

• By default, all sequences are displayed initially in their original configuration. Therefore the user does not have to go through all stages and can work on improving the initial MSA immediately.

• Users can also choose to start from the best solution from those submitted by the other advanced players. This enables players to work collaboratively to improve difficult MSAs.

• Users can modify the ancestor sequences reconstructed with our variant of the Fitch algorithm [22]. This allows advanced players to improve the score of an MSA if the ancestor calculated by our algorithm is sub-optimal (see section 'Video game scoring scheme’).

• Several levels of zoom of the MSA board are available, to give a global or local view of the game.

• A user can save their current configuration at any time and revert to it on demand (and not only the best one as in the classic version).

• A user can also submit their solution to our system at any time and still continue to play the same puzzle.

The advanced player (expert) version of Phylo is restricted to registered players who have completed at least 20 puzzles, and thus gained experience, with the classic version. As in most crowdsourcing applications, the number of advanced players is one to several orders of magnitude lower than the basic version. The reasons are that some players only play a few games before leaving, other players never register and finally some players prefer to play casual games rather than working on more sophisticated problems.

### Data sets

We evaluated Open-Phylo on MSAs of promoters regions of tumor suppressor genes: the P53 tumor suppressor protein, breast cancer type 1 susceptibility protein (BRCA1) and retinoblastoma protein (RB1). The sequences and initial Multiz alignments were downloaded from the UCSC Genome Browser [23].

These initial alignments were divided into smaller MSAs of 300 columns. Each of these MSAs was realigned with one of the four alignments programs used in this study (Multiz [14], MUSCLE [15], PRANK [16] or T-Coffee [17]) using the default alignment settings. The latter were the initial MSAs uploaded to the Open-Phylo web-user interface. All data (initial MSAs together with the MSAs improved with Open-Phylo) are available at [24].

### Video game scoring scheme

The casual and expert versions of the video game Phylo use the same scoring scheme. This is a simplified version of more realistic objective functions used to estimate the quality of an MSA. In our case, the scoring scheme for a given puzzle alignment must be evolutionarily realistic while being intuitive and fast to compute (as it is recomputed in real time every time the player modifies the alignment).

We made minor modifications to the scoring scheme to improve on that used in the first version of the casual game [5]. The Phylo interface displays a simplified and entertaining representation of an MSA instance with its associated phylogenetic tree. Each nucleotide is represented with a brick whose color indicates its type (adenine, cytosine, guanine or thymine). To evaluate a given alignment, the game infers ancestral nucleotides or gaps at each ancestral node of the phylogenetic tree using a maximum parsimony approach (the Fitch algorithm [22]), considering a gap as a fifth character, independently for each position. The scores for induced pairwise alignments, each evaluated using an affine gap cost model, are summed over all edges of the tree. To make the scoring intuitive, our scheme uses integer values (the score for a match is +1, for a mismatch -1, for a gap opening -4 and for a gap extension -1), which approximate those used by BLASTZ [25]. Compared to the value used in the original casual Phylo game [5], the gap opening score has been reduced in our new implementation. This change allows gamers to accommodate more gaps and it makes the game more entertaining while keeping the scoring realistic.

Because it infers ancestral nucleotides independently at each position, the original Fitch algorithm is not designed to accommodate an affine gap penalty model and may result in sub-optimal ancestral sequences, which would yield a pessimistic alignment evaluation. However, exact algorithms or better approximations are computationally more expensive [2,26], and we considered that the simplicity of our scoring method and its speed largely compensate for the slight accuracy loss. Nonetheless, we addressed this issue in the expert version and enabled users to modify the ancestor sequences (see section 'Advanced player (expert) version’). Therefore, advanced players can improve sub-optimal ancestors calculated by the game, and identify good MSAs that would be missed by the classical scoring algorithm.

Finally, our new version of Phylo also ignores gaps at the beginning and the end of each pairwise alignment. This modification enabled us to counter a basic strategy used in the first version of the casual Phylo game [5], which consisted in pushing all sequences to the left (or right) to minimize the number of gaps. While solutions using this technique often improve the score of the initial casual puzzle within the game, they rarely improve complete MSAs using more realistic objective functions. This new feature also made the game more challenging and thus entertaining.

### Objective function settings

In this study, we used version 3.8.31 of MUSCLE and version 9.03 of T-Coffee to calculate and score alignments. The alignments calculated with version 100303 of PRANK were scored with version 1.3.1 of GUIDANCE. The latter samples bootstrap neighbor joining trees to evaluate alignments. We chose to generate 50 bootstrapping trees, which seems to offer the best trade-off between the accuracy of the confidence score and running time.

## Abbreviations

bp: Base pair; MSA: Multiple sequence alignment.

## Competing interests

The authors declare that they have no competing interests.

## Authors’ contributions

DK designed and implemented the Open-Phylo interface and server. AK designed and implemented the Phylo casual video game. DB analyzed the data. QZ designed and implemented the Phylo expert interface. AH prepared the data. EZ participated in the design and implementation of the Open-Phylo interface, Arthur K participated in the implementation of the Phylo casual video game (in part while he was affiliated with the Nokia Research Center). LS contributed to the design of the Phylo casual video game. MB designed the research and wrote the manuscript. JW designed the research, implemented the server, analyzed the data and wrote the manuscript. All authors read and approved the final manuscript.

## Supplementary Material

Additional file 1: Table S1 Scores for the alignments of the 338 blocks used in the benchmark. Each row is for an expert block aligned using one of the four programs (Multiz, MUSCLE, PRANK or T-Coffee). Each column shows the scores obtained using one of the four scoring functions (Ancestor, MUSCLE, GUIDANCE or T-Coffee) for the three types of alignment (the initial alignment, the best alignment obtained with the Phylo Classic version and the best alignment obtained by the Phylo Expert version). Highlighted cells show scores that improve the initial score. Click here for file
